# Detection for gene-gene co-association via kernel canonical correlation analysis

**DOI:** 10.1186/1471-2156-13-83

**Published:** 2012-10-08

**Authors:** Zhongshang Yuan, Qingsong Gao, Yungang He, Xiaoshuai Zhang, Fangyu Li, Jinghua Zhao, Fuzhong Xue

**Affiliations:** 1 Department of Epidemiology and Health Statistics, School of Public Health, Shandong University, Jinan, 250012, China; 2 Berlin Institute for Medical Systems Biology, Max-Delbrück-Center for Molecular Medicine, 13125, Berlin, Germany; 3 CAS-MPG Partner Institute for Computational Biology, Shanghai Institutes for Biological Sciences, Chinese Academy of Sciences, Shanghai, 200031, China; 4 Key Laboratory of Computational Biology, CAS-MPG Partner Institute for Computational Biology, Chinese Academy of Sciences, Shanghai, 200031, China; 5 MRC Epidemiology Unit, Institute of Metabolic Science, Addenbrooke’s Hospital, Cambridge, UK

**Keywords:** Genome-wide association study (GWAS), Gene-gene co-association, Gene-gene interaction (GGI), Kernel canonical correlation analysis (KCCA)

## Abstract

**Background:**

Currently, most methods for detecting gene-gene interaction (GGI) in genomewide association studies (GWASs) are limited in their use of single nucleotide polymorphism (SNP) as the unit of association. One way to address this drawback is to consider higher level units such as genes or regions in the analysis. Earlier we proposed a statistic based on canonical correlations (CCU) as a gene-based method for detecting gene-gene co-association. However, it can only capture linear relationship and not nonlinear correlation between genes. We therefore proposed a counterpart (KCCU) based on kernel canonical correlation analysis (KCCA).

**Results:**

Through simulation the KCCU statistic was shown to be a valid test and more powerful than CCU statistic with respect to sample size and interaction odds ratio. Analysis of data from regions involving three genes on rheumatoid arthritis (RA) from Genetic Analysis Workshop 16 (GAW16) indicated that only KCCU statistic was able to identify interactions reported earlier.

**Conclusions:**

KCCU statistic is a valid and powerful gene-based method for detecting gene-gene co-association.

## Background

Genome-wide association studies (GWASs), which may involve a large number of single nucleotide polymorphisms (SNPs) on many individuals, are widely used to identify genetic variants underlying complex diseases or other types of traits. Although a primary interest in a GWAS is to identify SNPs associated with a trait of interest, it is important to consider the associate genes and their co-association as well. One form of co-association is epistasis, which was introduced approximately 100 years ago and generally defined as interactions among genes [1]. These are linked to gene-gene interactions (GGIs) which are often characterized to be functional, compositional and statistical [2]. The statistical definition was given by Fisher [3] and developed further by Cockerham [4] and Kempthorne [5], whereby the effect of GGIs is treated as deviation from additive genetic effects of single genes [6].

Methods to detect GGIs on the basis of the statistical definition include but are unlimited to logistic regression, multifactor dimensionality reduction [7], linkage disequilibrium (LD)-based [8,9] and entropy-based statistics [10,11], together with others implemented in PLINK[12], Tuning ReliefF [13], Random Jungle[14], BEAM[15] and BOOST[16]. However, most of these consider SNP as the unit of association, which has limitations and are insufficient for interpretation of GGI [17] which calls for consideration of higher level units such as genes or regions in the analysis. Gene-based analysis can account for multiple independent functional variants within genes with a potential increase of power to identify GGI. Earlier, Peng et al. [17] proposed a gene-based statistic (CCU statistic) for detecting gene-gene co-association based on canonical correlation analysis (CCA) in a case–control study, which was defined as joint effect of genes contributing to a binary trait and proved to have good performance on detecting gene-gene co-association or GGI. However, CCA can only detect linear correlation, and may be inappropriate for genomic data containing nonlinear structure. Recent years have witnessed considerable work and successes on kernel CCA (KCCA) as a nonlinear generalization of the classical CCA in machine learning, face recognition, data classification [18-20], and notably genomic data analysis by Yamanishi et al. [19]. We here construct a kernel CCU (KCCU) statistic for detecting gene-gene co-association and evaluate its performance via simulations and data analysis.

## Methods

### CCA

CCA is a classical multivariate method which concerns about linear dependencies between sets of variables. Let *X*_*i*_, *Y*_*i*_ (i = 1, . . ., m) denote samples of measurements on m objects, We assume the data to be column centred. Let *A* be any *m × n* matrix then *L*(*A*) = {*Aα*|*α* ∈ *R*^*n*^} will be referred to as the column-space and *L*(*A*^*T*^) = {*A*^*T*^*α*|*α* ∈ *R*^*m*^} the row-space of *A*. The aim of canonical correlation analysis is to determine vectors *ν*_*j*_ ∈ *L*(*X*^*T*^) and *ω*_*j*_ ∈ *L*(*Y*^*T*^) such that *a*_*j*_ = *Xν*_*j*_ and *b*_*j*_ = *Yω*_*j*_ are maximally correlated. $cor\left(a_{j},b_{j}\right)=\frac{〈a_{j},b_{j}〉}{\left|\left|a_{j}\right|\right|\cdot \left|\left|b_{j}\right|\right|}$ with 〈〉 indicating inner product. Usually, this is formulated as a constrained optimization problem $\begin{array}{l} \arg\max{\nu_{j}}^{T}X^{T}Y\omega_{j}\\ \nu_{j}\in L(X^{T}),\omega_{j}\in L(Y^{T}) \end{array}$ subject to ${\nu_{j}}^{T}X^{T}X\nu_{j}={\omega_{j}}^{T}Y^{T}Y\omega_{j}=1$ which yields the first pair of canonical vectors (*ν*_1_, *ω*_1_) and *a*_1_ = *Xν*_1_, *b*_1_ = *Yω*_1_ are the corresponding canonical variates and their correlation is called the maximum canonical coefficient. Pairs of canonical vectors (*ν*_*j*_, *ω*_*j*_) can be recursively defined by maximizing similar expression and keeping subsequent variates orthogonal to those previously obtained. CCA can be interpreted as constructing pairs of factors from *X* and *Y*, respectively by linear combination of the variables involved, as a way to account for linear dependencies between sets of variables.

### KCCA

KCCA generalizes CCA as follows: Objects *x*_*i*_ and *y*_*i*_ are first mapped to some Hilbert spaces *H*_*x*_ and *H*_*y*_ through mapping Φ_*x*_(.) and Φ_*y*_(.), CCA is then performed on images {Φ_*x*_(*x*_*i*_)}_*i* = 1_^*m*^ and {Φ_*y*_(*y*_*i*_)}_*i* = 1_^*m*^. Let K_*x*_ and K_*y*_ denote *m × m* kernel inner product matrices (also known as kernel gram matrices), constructed element-wise as ${\left({Κ}_{x}\right)}_{\mathrm{ij}}=〈\Phi_{x}\left(x_{i}\right),\Phi_{x}\left(x_{j}\right)〉$ and ${\left({Κ}_{y}\right)}_{\mathrm{ij}}=〈\Phi_{y}\left(y_{i}\right),\Phi_{y}\left(y_{j}\right)〉$. Analogous to CCA, the aim of KCCA is to find canonical vectors in terms of expansion coefficients *α*_*j*_, *β*_*j*_ ∈ *R*^*m*^ as a constrained optimization problem $\begin{array}{l} \arg\max{\alpha_{j}}^{T}{Κ}_{X}{Κ}_{Y}\beta_{j}\\ \alpha_{j},\beta_{j}\in R^{m} \end{array}$ subject to ${\alpha_{j}}^{T}{Κ}_{X}{Κ}_{X}\alpha_{j}={\beta_{j}}^{T}{Κ}_{Y}{Κ}_{Y}\beta_{j}=1$.

Explicit form for the mapping Φ_*x*_(.) and Φ_*y*_(.) are not always required but the kernel K_*x*_ and K_*y*_ need to be fixed. Common kernel functions include linear, polynomial, radial basis function (RBF), sigmoid [21], identical-by-state and weighted identical-by-state kernels [22]. It is worthwhile to note that these kernel functions generally have similar performance with parameters that are appropriately chosen.

### Test statistic

Strategy analogous to CCU statistic was used to construct the KCCU statistic except that the maximum kernel canonical coefficient of the two genes, rather than the maximum canonical coefficient, was taken as a measure of gene-gene co-association in cases and controls. Let genotyped data of case–control study be (*X*_1_^*D*^, *X*_2_^*D*^, …, *X*_*P*_^*D*^) and (*Y*_1_^*D*^, *Y*_2_^*D*^, …, *Y*_*q*_^*D*^) for gene A and gene B for cases, and (*X*_1_^*C*^, *X*_2_^*C*^, …, *X*_*P*_^*C*^) and (*Y*_1_^*C*^, *Y*_2_^*C*^, …, *Y*_*q*_^*C*^) for controls. The maximum kernel canonical coefficient *κr*_*D*_ between (*X*_1_^*D*^, *X*_2_^*D*^, …, *X*_*P*_^*D*^) and (*Y*_1_^*D*^, *Y*_2_^*D*^, …, *Y*_*q*_^*D*^) obtained through KCCA could be considered as a measurement of gene-based gene–gene co-association in cases, and *κr*_*C*_ between (*X*_1_^*C*^, *X*_2_^*C*^, …, *X*_*P*_^*C*^) and (*Y*_1_^*C*^, *Y*_2_^*C*^, …, *Y*_*q*_^*C*^) be a measurement of gene–gene co-association in controls. The transformation analogous to Fisher’s simple correlation coefficient transformation was done to *κr*_*D*_ and *κr*_*C*_, i.e. $\kappa z_{D}=\frac{1}{2}\left(\log\left(1+\kappa r_{D}\right)-\log\left(1-\kappa r_{D}\right)\right)$ and $\kappa z_{C}=\frac{1}{2}\left(\log\left(1+\kappa r_{C}\right)-\log\left(1-\kappa r_{C}\right)\right)$.

The KCCU statistic for detecting statistical significance of the difference of gene-based gene-gene co-association between cases and controls can be defined as $U=\frac{\kappa z_{D}-\kappa z_{C}}{\sqrt{\mathrm{var}\left(\kappa z_{D}\right)+\mathrm{var}\left(\kappa z_{C}\right)}}$, which is approximately *N*(0,1). With the difficulty in obtaining an explicit form for var(*κz*_*D*_) and var(*κz*_*C*_), a bootstrap procedure was employed. Seeing that the performance of kernel methods strongly relates to the choice of kernel functions and their parameters, we chose the RBF kernel owing to its flexibility in parameter specification [23]. In general, two approaches are popular: 1. via empirically assigning candidate values for the parameter(s) involved subject to a learning algorithm for the best performance; 2. via some cross-validation procedure. Both are computer intensive [24].

### Data simulation

**Figure 1:**
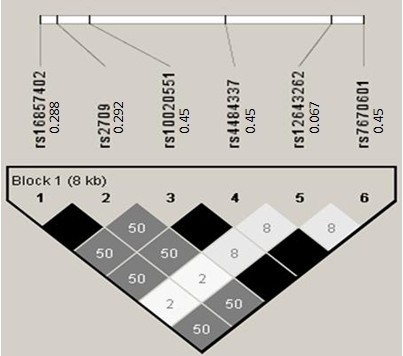
**Pairwise r**^**2 **^**among the six SNPs in the first region.** The six SNPs are rs16857402, rs2709, rs10020551, rs4484337, rs12643262, and rs7670601. The values to the right of the 6 dbSNP IDs (rs# IDs) are the corresponding minor allele frequencies.

**Figure 2:**
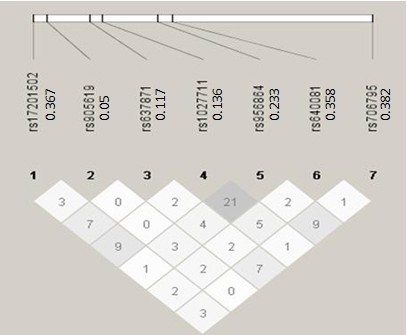
**Pairwise r**^**2 **^**among the seven SNPs in the first region.** The seven SNPs are rs17201502, rs905619, r637871, rs1027711, rs956864, rs640081, and rs706795. The values to the right of the seven dbSNP IDs (rs# IDs) are the their minor allele frequencies.

Simulation studies were conducted to assess the performance of KCCU relative to CCU under both the null (*H*_0_) and alternative hypotheses (*H*_1_), which were based on the HapMap data in the following steps:

Step 1. Phased haplotype (Phases 1 & 2 of CEU) data were downloaded from the HapMap web site (http://snp.cshl.org) on two unlinked genome regions for generating the simulated genotypes. The *GNPDA2* region is on Chr 4: 44401210.44410098 involving six SNPs while *FAIM2* region is on Chr 12: 48571829.48583937 involving seven SNPs. Their LD patterns where shown in Figures 1 and 2 together with pairwise *r*^*2*^.

Step 2. Based on data above, large samples with 100, 000 cases and 100, 000 controls were generated using software gs2.0 [25] under a two-locus interaction multiplicative effects model (see Additional file 1), treating the 2^nd^ SNP of the first region and the SNP of the other as the causal variants and they were removed in the simulation to assess gene-gene co-association. The interaction odds ratio was set as 1.0 under *H*_0_ and 1.1, 1.2, 1.3, 1.4, 1.5 under *H*_1_. The SNPs in the regions were coded according to an additive genetic model. To further investigate the performance on causal SNPs with respect to minor allele frequency and LD, different SNP pairs from the two gene regions were defined as the casual variants.

Step 3. From the remaining SNPs, simulated data were sampled and CCU and KCCU performed under various sample sizes *N* (*N*/2 cases and *N*/2 controls, *N* = 1000…5000) with R package kernlab (http://cran.r-project.org/web/packages/kernlab/index.html). 500 simulations were repeated each with a significant level of 0.05.

### Applications

The proposed KCCU statistic was applied to rheumatoid arthritis (RA) data from GAW16 Problem 1, consisting of 2,062 Illumina 550k SNP chips from 868 RA patients and 1,194 normal controls collected by the North American Rheumatoid Arthritis Consortium [26]. Three genes (*C5*, *ITGAV*, and *VEGFA*) on three different chromosomes were selected to detect gene-gene co-association in this work, involving eight, eight and four SNPs, respectively. Logistic regression test and the CCU statistic were also used. For each pair of genes, the statistic which yielded the minimum p value was recorded from all pairs of SNPs one on each gene. The significance of the statistic was compared to its empirical distribution generated from 1,000 permutations by permuting case–control labels [27] which is relatively easy compared to the “BY” method [28] for multiple testing adjustment.

## Results

### Simulation

Shown in Table 1 are simulation results under *H*_0_. The KCCU statistic is normally distributed according to the one sample Kolmogorov-Smirnov test with the type I error rates of KCCU statistic being close to given nominal value (α = 0.05) for different sample sizes. This indicates that the proposed statistic performs well under the null hypothesis.

**Table 1:** Performance of CCU and KCCU under the null hypothesis

**Sample size**	**CCU**		**KCCU**	
	**Type I Error**	**Normality Test (D)**	**Type I error**	**Normality Test (D**)
1000	0.052	>0.55	0.049	>0.55
2000	0.051	>0.55	0.054	>0.55
3000	0.056	>0.55	0.052	>0.55
4000	0.048	>0.55	0.051	>0.55
5000	0.053	>0.55	0.050	>0.55

D, Kolmogorov-Smirnov D test.

**Figure 3:**
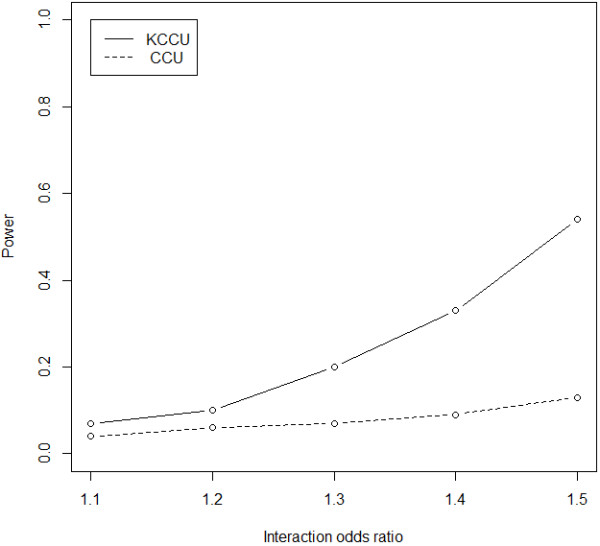
Power of CCU and KCCU statistics given different interaction odds ratios and a sample size of 3,000.

**Figure 4:**
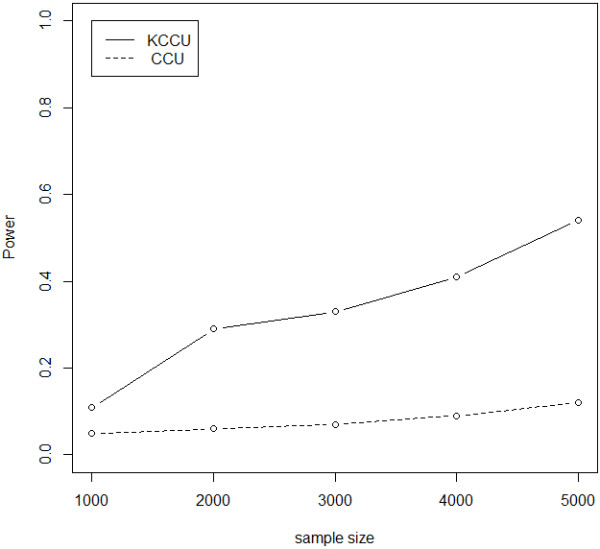
Power of CCU and KCCU statistics given an interaction odds ratio of 1.4 and different sample sizes.

**Figure 5:**
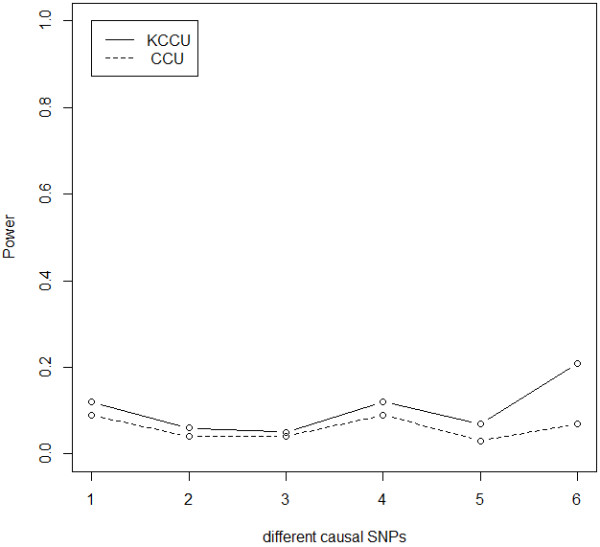
Power of CCU and KCCU statistics when SNP pairs from two regions are defined as casual variants at an interaction odds ratio of 1.3 and a sample size of 3,000.

Results on various interaction odds ratios and a sample size of 3,000 are shown in Figure 3, as with different sample sizes with an interaction odds ratio of 1.4 in Figure 4. It is clear that power of KCCU is a monotonically increasing function of sample size and interaction odds ratio. Figure 5 shows results with different SNP pairs defined as causal SNPs with an interaction odds ratio of 1.3. The power of KCCU statistic was higher than that of CCU statistic. Power as a function of interaction odds ratio for different sample size is provided as Additional file 1.

### Application

The performance of logistic regression test, CCU and KCCU statistics on pair-wise gene-gene co-association of three genes is shown in Table 2, which also contains results on the Gaussian RBF kernels with various parameter values (σ=0.05, 0.5, 5 and 50). Through KCCU the three genes were shown to have co-association with each other at significance level 0.05 regardless the parameter value, in contrast to the CCU statistics showing no significant co-association and none of the SNP pairs were significant under logistic regression test with correction for multiple testing.

**Table 2:** **P-values of gene-gene co-association among *C5*, *ITGAV *and *VEGFA***

**Co-association**		***C5-ITGAV***	***C5-VEGFA***	***ITGAV-VEGFA***
Logistic regression		0.1015	0.1425	0.1840
CCU		0.5387	0.5325	0.8317
KCCU	σ=0.05	<0.001^*^	<0.001^*^	<0.001^*^
	σ=0.5	<0.001^*^	<0.001^*^	<0.001^*^
	σ=5	<0.001^*^	<0.001^*^	<0.001^*^
	σ=50	<0.001^*^	<0.001^*^	<0.001^*^

*significant at level 0.05.

## Discussion

We have extended the CCU statistic to a new statistic KCCU, which can extract nonlinear correlation between two genes. Simulation studies show that both CCU and KCCU statistics performed well under null hypothesis with KCCU being more powerful than CCU with respect to significant level, sample size and relative risk. As results vary with user-defined kernel parameter, various parameters were used (the bandwidth parameter in RBF kernel) to RA data in GAW16 Problem 1, showing that the logistic regression test and CCU statistic failed to detect any interaction but KCCU statistics identified the pair-wise interactions among the three genes under various parameters. The interaction between *ITGAV* and *VEGF* genes has been identified by a rank method [29]. As suggested by a reviewer, it is critical to consider time-efficiency in genome-wide association studies to make the proposed methods practical. In our case, the computing time as required for KCCU was about 2.5 times slower than CCU, but nevertheless will still be feasible with the development as well as the extensive applications of multiprocessor and multithreading computational technique.

A reviewer has also suggested us to reiterate the relationship between gene-gene co-association and GGI which is readily available. GGI generally refers to the synergetic or antagonistic effect of two genes in addition to the summation of their independent effects on an outcome. To represent the interaction between two genes A and B in a case–control association study, a product term is customarily added to the logistic regression model *Logit*(*P*) = *β*_0_ + *β*_1_*A* + *β*_2_*B* + *γA* × *B* so that *γ* reflects both the direction and size of the interaction. This model implicitly assumes that gene A and gene B are independent so as to infer interaction (*γ*). However, it might well be that genes are correlated with each other in genetic networks to contribute to disease susceptibility, so the independence assumption is rarely ratified. Gene-gene co-association extends the concept of GGI in that it describes the generic joint distribution of two gene effects on disease or trait without assuming either independence or linear relationship. Here the measurement of the co-association between genes is based on the correlation between genes (such as CCU statistic and KCCU statistic), provides a measure of the contribution of two genes. As for two unlinked genes, their relationship can be described as either co-association or interaction. The reviewer has also brought to our attention to earlier work by Song and Nicolae [30] on imposing natural restrictions for the parameter space and discussion on the definition of “no interaction” between two unlinked loci as two loci being independent conditioned on the subject having the disease. In this paper, the null hypothesis of the proposed test is that there is no gene-gene co-association (i.e. GGI for two unlinked genes), the data under the null hypothesis are generated from the gs software with the interaction odds ratio parameter to be one.

Several issues remain to be resolved: the uncertainty to set the kernel function with appropriate parameters for each data, the undesirable performance of both CCU and KCCU with small interaction odds ratio (e.g. 1.1), and the possible failure of maximum kernel canonical correlation coefficient to represent gene-gene co-association.

## Conclusions

KCCU statistic is a valid and powerful gene-based method for detecting gene-gene co-association compared to CCU and logistic regression test. Further work is needed to make its use in GWAS more practical.

## Competing interests

The authors declare that they have no competing interests.

## Authors’ contributions

ZSY, QSG, YGH, XSZ, FYL, JHZ and FZX conceptualized the study, acquired and analyzed the data and prepared for the manuscript. All authors approved the final manuscript.

## Supplementary Material

Additional file 1 Two-locus interaction multiplicative effects model. Click here for file
