# Supplementary material for: Detection for gene-gene co-association via kernel canonical correlation analysis
Source: BMC Genet. 2012 Oct 8;13:83. doi: 10.1186/1471-2156-13-83 (PMC3506484; doi:10.1186/1471-2156-13-83)
Supplement: Additional file 1 — Two-locus interaction multiplicative effects model. [file 1471-2156-13-83-S1.docx]

**Supplementary materials**

**Two-locus interaction multiplicative effects model**

The gs program has implemented nine commonly used models in the literature. For each of these nine models, users only need to specify the population prevalence *p*, and genotype odds ratio(s) $1+\theta$for each locus, then the program can automatically calculate the penetrance table. Among the nine commonly used models is the model of two-locus interaction multiplicative effects, and can be shown in the following table , where $\alpha$is the baseline value which indicates the odds of disease when the two loci do not carry any disease alleles. For example, when the genotype combination is Aabb, the odds of disease is $\alpha$, and it changes to $\alpha(1+\theta)$ when the genotype combination is AaBb due to the disease allele B, and will be $\alpha(1+\theta)^{2}$ when the genotype combination is AaBB. That is, the odds of disease will multiply $1+\theta$ once the genotype combination increases one disease allele.

|  | bb | Bb | BB |
| --- | --- | --- | --- |
| aa | $\alpha$ | $\alpha$ | $\alpha$ |
| Aa | $\alpha$ | $\alpha(1+\theta)$ | $\alpha(1+\theta)^{2}$ |
| AA | $\alpha$ | $\alpha(1+\theta)^{2}$ | $\alpha(1+\theta)^{4}$ |

# Reference

# Li W, Reich J: A complete enumeration and classification of two-locus disease models. Hum Hered. 2000, 50(6): 334-349


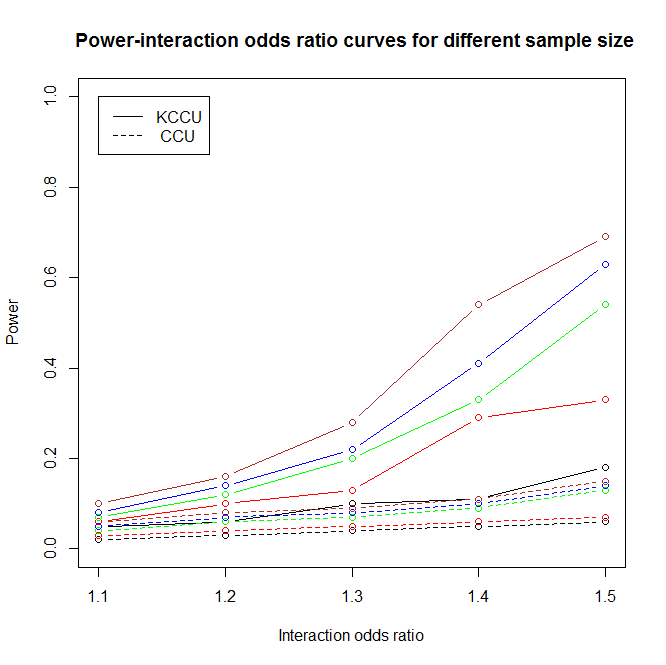


Figure S1 Power-interaction odds ratio curves for different sample size, black curve for sample size 1000, red for sample size 2000, green for sample size 3000, blue for sample size 4000, orange for sample size 5000.
